# A Nomogram Model for Mortality Risk Prediction in Pulmonary Tuberculosis Patients Subjected to Directly Observed Treatment Shortcourse (DOTS)

**DOI:** 10.1155/2022/1449751

**Published:** 2022-12-16

**Authors:** Yi Xie, Jing Han, Weili Yu, Zhili Hou, Zhen Wan

**Affiliations:** ^1^Haihe Hospital, Tianjin University, Tianjin 300350, China; ^2^Tianjin Institute of Respiratory Diseases, Tianjin 300350, China

## Abstract

We analyzed the risk factors of mortality for patients with pulmonary tuberculosis under the Directly Observed Treatment Shortcourse (DOTS) and established a predictive nomogram for the risk of mortality. The retrospective cohort analysis was conducted on the treatment outcomes of 11207 tuberculosis patients in the tuberculosis management information system in Tianjin from 2014 to 2019. Based on the multivariable unconditional logistic regression, we analyzed the risk factors of mortality in patients with pulmonary TB and established the death risk prediction nomogram. We further applied cross-validation and the receiver operating characteristic (ROC) curve to explore the efficiency of the nomogram. There were 10,697 patients in the survival group and 510 in the mortality group who had successfully initiated DOTS, and the mortality rate was 4.55%. Multivariable logistic regression analysis showed that age, male, relapse cases, first sputum positivity, patient delay, and HIV-positive were independent risk factors for pulmonary TB death. The calibration curve shows that the average absolute error between the predicted mortality risk and the actual death risk is 0.003. The ROC curve shows that the area under the curve where the line-up model predicts the risk of death is 0.816 (95% CI: 0.799∼0.832). The nomogram model based on independent risk factors of mortality in TB patients shows good discrimination and accuracy, with potentially high clinical value in screening patients with a high risk of death, which could be useful for setting the interventional strategies in patients with tuberculosis who had successfully initiated DOTS.

## 1. Introduction

Tuberculosis (TB) is a communicable disease that is a major cause of ill health. Tuberculosis is the cause of death of a single infectious disease, second only to COVID-19. The global death toll of tuberculosis will rise from 1.4 million in 2019 to 1.5 million in 2020 and then to 1.6 million in 2021 [1]. In China, there are about 900,000 new cases each year, ranking third among the 30 countries with a high burden of TB [2]. Since 1992, China has implemented the Directly Observed Treatment Shortcourse (DOTS) nationwide and achieved a certain success. However, the incidence of pulmonary tuberculosis in the infectious disease epidemic network reporting system still ranks second in class A and B infectious diseases and the third leading cause of death from major infectious diseases [3]. Relevant studies have shown that more than half of the deaths occur in the first 2 months of TB treatment. It is urgent to explore and establish a mortality risk prediction model for TB patients during shortcourse supervised treatment [4].

In this retrospective cohort study, we analyzed the clinical material of patients with pulmonary tuberculosis who had successfully initiated DOTS, explored the risk factors associated with supervised treatment mortality, and established a nomogram model for individualized prediction of the risk of death. Furthermore, this model will improve the anticipative ability of medical staff to reduce the mortality of pulmonary tuberculosis during treatment.

## 2. Materials and Methods

### 2.1. Patients

**Figure 1:**
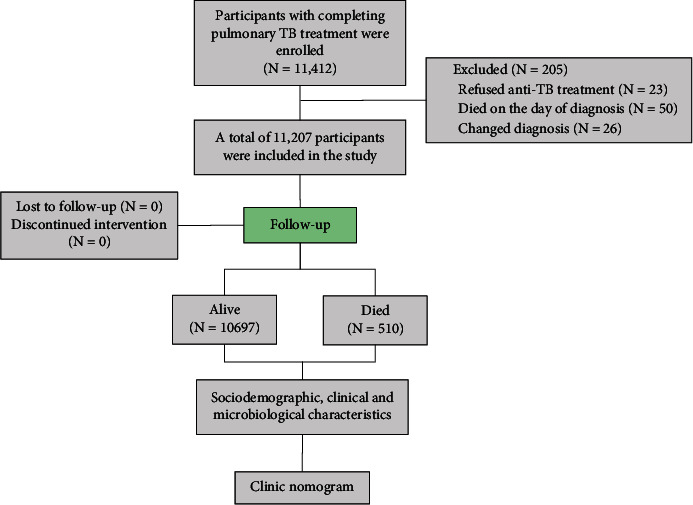
Flow diagram for participant inclusion and exclusion.

From January 1, 2014, to December 31, 2019, a registration-based cohort study was conducted with resident populations admitted to the municipal TB-designated hospitals in Tianjin who were diagnosed with pulmonary tuberculosis (11,412). Pulmonary TB was diagnosed according to the TB diagnostic criteria (WS288-2008) and guidelines for the diagnosis and treatment of tuberculosis by the Chinese Society for Tuberculosis, Chinese Medical Association [5]. The diagnosis was based on etiological examination (including bacteriology and molecular biology), combined with epidemiological history, clinical manifestations, chest images, relevant auxiliary examinations, and differential diagnosis. In this study, 11,207 patients were included (Figure 1), while 205 patients were excluded. Among the excluded, 23 cases refused anti‐TB treatment, 50 cases died on the day of diagnosis, 26 cases had their diagnosis changed, and 106 cases were lost to follow-up. For the 106 patients lost to follow-up, the average age was 47.52 ± 18.24 years, 72 patients (67.92%) were male, and 84 patients (79.24%) were initially treated. Furthermore, it was not statistically significant (*P* > 0.05) in comparison to participants who were retained in DOTS. A total of 50 patients died on the day of diagnosis, whose average age was 64.92 ± 20.22 years, 42 patients (84%) were male, and 41 patients (82%) were new cases. There was no statistically significant (*P* > 0.05) in comparison to the 510 patients who died during DOTS. In total, the two groups presented no significant differences.

### 2.2. Data Sources

The basic information about patients with TB included in the study was collected by querying the tuberculosis information management system in China and the hospital information system. All information mainly includes the following content: sex, age, ethnicity, case classification (new cases and relapse cases), results of first sputum smear microscopy, time from symptom appearance to consultation, time from consultation to diagnosis, pulmonary cavity, human immunodeficiency virus (HIV) laboratory results, first-line antiTB drug resistance, the prognosis of treatment, and other data. Patient delay is >14 days from symptom appearance to consultation, while the diagnosis delay is >14 days from consultation to diagnosis [6].

According to the requirements of the “Law of the People's Republic of China on the Prevention and Control of Infectious Diseases” and the “Guidelines for the Implementation of China's Tuberculosis Prevention and Control Program,” network reporting of TB patients should be conducted. In China, it is compulsory that all TB cases should be directly reported to the Ministry of Health of The People's Republic of China. The medical staff of tuberculosis prevention and control should register the patients diagnosed with tuberculosis within 24 hours. Supervised chemotherapy and follow-up management were implemented by TB prevention and control personnel. All the patients included in the study were followed up by telephone or home visit during DOTS, and there were no missing data. According to the requirements of the guidelines, all patients should regularly visit designated TB hospitals for follow-up laboratory examinations, including sputum smear, sputum culture, chest imaging, blood routine, liver and kidney function, and other tests. The outcome of the study was the death of the patients during the follow-up period, including all-cause mortality. The Guidelines required that the supervised treatment periods were 6 months, 7 months, 8 months, 9 months, and 10 months. The diagnosis, treatment, supervision, follow-up management, and other information on pulmonary TB should be registered in the tuberculosis information management system in China. The medical staff should strictly implement modern tuberculosis control strategies. The total duration of participant follow-up was from January 1, 2014, to December 31, 2020. The median time of follow-up was 6 months in this study. The follow-up deadline was December 31, 2020.

### 2.3. Statistical Analysis

We used SPSS 19.0 software for the statistical analysis of data. The numerical variables with normal distributions were expressed as $\bar{x}$ ± *s*, and the comparisons between the two groups were performed with an independent-samples *t*-test. Enumeration data were expressed as cases (%), and the *χ*^2^ test was used for comparison between the two groups. Multivariable unconditional logistic regression analysis was performed by the forward and backward stepwise regression to determine independent risk factors using death during DOTS as the outcome of patient prognosis. The independent risk factors were imported into R software (R2.12.1), and the RMS program package was applied to establish a prediction model of the nomogram. A 10-fold cross-validation procedure (iteration number = 1000 times) was used to internally validate the nomogram model. The ROC curve of the nomogram model for predicting the risk of death was plotted, and the area under the curve (AUC) and Brier score were calculated to evaluate the calibration appearance of the model. The mean (standard deviation [SD], range) of AUCs from the 1000 iterations was reported. The test level *α* was set to 0.05 (two-sided) to calculate the nomograph score of each variable according to the regression coefficient of the variable (*β*) and variable value range (distance).

## 3. Results

### 3.1. Subject Characteristics

A total of 11,207 patients' clinical characteristics were statistically analyzed and shown in Table 1. The average age of all patients was 49.61 ± 19.99 years, ranging from 12 to 110, and 7,786 patients (69.47%) were male. According to the treatment classification, there were 9,107 patients (81.26%) and were initially treated. According to the lab reports, 6,413 patients (57.22%) were first sputum bacteria positivity, 2,624 patients (23.41%) had cavities in the lungs, 20 patients (0.18%) were HIV positive, 1,469 patients (13.11%) had multidrug-resistant (MDR) according to drug sensitivity results, and 9,738 cases (86.89%) are nondrug resistance. In this study, 510 patients (4.55%) died during DOTS, of which 51.76% (264/510) died during the first 2 months of the antiTB treatment.

We divided all patients into death and alive status and analyzed 10 factors. We found that age, sex, case classification, results of first sputum smear microscopy, HIV status, and patient delay were significantly different (*P* < 0.05) (Table 1).

### 3.2. Death Risk Factors in Pulmonary TB Patients under the DOTS Strategy

Multivariable unconditional logistic regression analysis was performed with pulmonary TB patients' death under the DOTS strategy as the dependent variable and the remaining parameters as independent variables. The results showed that male (OR = 1.915, 95% *CI*: 1.512∼2.425), advanced age (OR = 1.069, 95% CI: 1.062∼1.076), relapse cases (OR = 1.297, 95% CI: 1.052∼1.599), first sputum positivity (OR = 1.711, 95% CI: 1.388∼2.110), patient delay (OR = 1.286, 95% CI: 1.053∼1.571), and HIV-positive (OR = 4.497, 95% CI: 1.235∼16.372) were independent death risk factors in pulmonary TB patients (Tables 1–3).

### 3.3. Predictive Nomogram for the Risk of Death

**Figure 2:**
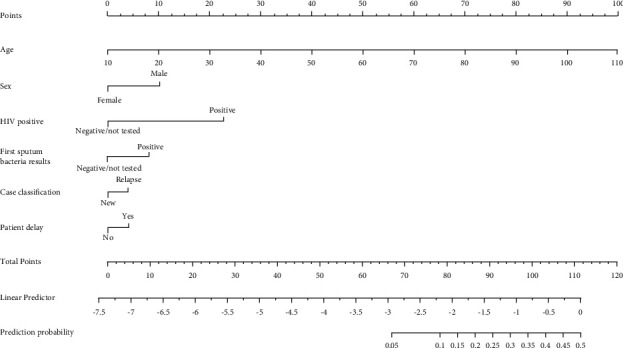
A nomogram predicting the risk of mortality among pulmonary tuberculosis patients under the DOTS strategy. (The value of each variable was given a score on the point scale axis. A total score could be easily calculated by adding every single score, and by projecting the total score to the lower total point scale, we were able to estimate the probability of pulmonary tuberculosis.).

In our study, the nomogram showed that the risk of death for pulmonary TB patients increased when patients were with advanced age, male, relapse case, first sputum positivity, patient delay, and HIV-positive (Figure 2 and Table 4).

### 3.4. Verification of the Nomogram

**Figure 3:**
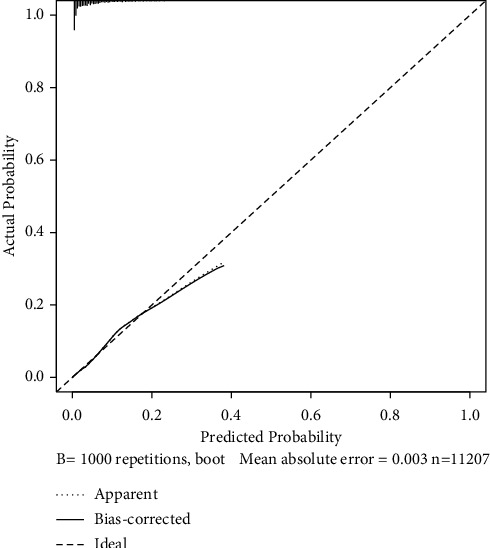
The calibration curves of the nomogram for the risk of mortality. (The *x*-axis represents the nomogram-predicted probability, and the *y*-axis represents the actual probability. A perfect prediction would correspond to the 45°black dashed line. The black dotted line represents the entire cohort (*n* = 11,207), and the black solid line is bias-corrected by bootstrapping (*B* = 1000 repetitions), indicating observed nomogram performance.

**Figure 4:**
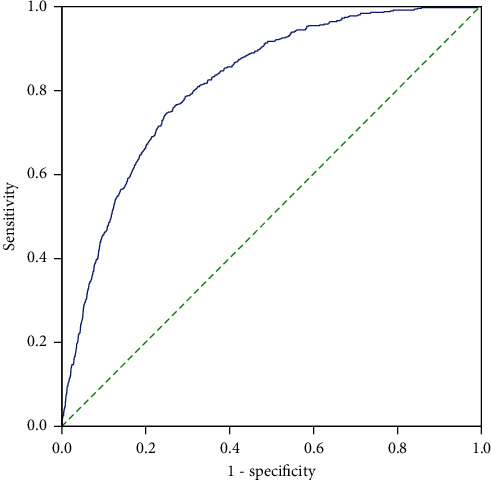
The ROC curve and AUC of the nomogram for the risk of mortality. (The blue solid line represents the nomogram-predicted probability. The reference axis would correspond to the 45°green solid line).

Predictive models with nomograms integrating all factors affecting mortality in pulmonary tuberculosis patients are shown in Figure 3. The results showed that the mean absolute error between the nomogram-predicted probability and the actual probability was 0.003, indicating the consistency of the model. The mean (SD, range) of AUC for the predictive mortality was 0.816 (0.030, 0.717∼0.879), which reflected the good discrimination ability of the model (Figure 4). The Brier score was 0.040, which reflected good predictive calibration ability.

## 4. Discussion

TB outbreak posed a huge social burden. It is shown that a death survey among pulmonary TB patients under DOTS, with predictors, further explored, is beneficial to reducing mortality and improving the prognosis outcome. Follow-up data of 11,207 pulmonary TB patients under DOTS in Tianjin from 2014 to 2019 were retrospectively analyzed. 510 patients died with a mortality of 4.55%, which was higher than those in Guangzhou (2.7%), in the United States (2.7%), and in New York (2%) but lower than those in Nigeria (16.6%), Ethiopia (12.71%), and India (6%) [8–13]. Our results show that 51.76% (264/510) of the deaths occurred in the first two months of intensive antituberculosis treatment, which is consistent with the results of previous studies [4]. Multivariable analysis indicated that independent risk factors for death among patients with pulmonary TB under the DOTS strategy included advanced age, male, HIV positivity, first sputum positivity, relapse cases, and patient delay. In a meta-analysis [14], factors such as age, sex, lack of sputum conversion at two months of treatment, and HIV affected the success of TB treatment.

The nomogram provided a simpler and more convenient method of visualizing the results from logistic regression analysis through some graphical notation, allowing for calculating the probability of events separately [15]. Compared with the traditional method, the nomogram considers more predictors and is flexible to apply. It is widely used in research as a predictive model of diseases such as cirrhotic patients with upper gastrointestinal bleeding, gastric cancer with lymph node metastasis, and multidrug resistant pulmonary tuberculosis (MDR-TB) [16–18]. Since 1992, the DOTS strategy has been gradually implemented in China for the treatment and management of pulmonary tuberculosis patients, and the sputum negative conversion rate and patient cure rate at the end of the intensive phase are used as the main assessment indicators of the effect of chemotherapy, without considering the effect of multiple factors on DOTS strategy and patient death. However, there have been no reports on the establishment of an individual model to predict the risk of death in patients with tuberculosis under DOTS with clinical characteristics. Based on the inrdependent prognostic factors of death of pulmonary tuberculosis patients, this study established the first forecast model of the death risk of patients with tuberculosis in China, which can realize the individual prediction and can calculate the mortality risks of each tuberculosis patient according to the score in the model. It will help the medical personnel of tuberculosis to directly identify the patients with high mortality risk who had successfully initiated DOTS according to different state levels of various factors.

According to the results of our study, sex is an independent predictor of mortality in TB patients with a 1.915-fold higher risk of mortality in males than in females, which is consistent with previous studies [19]. Males will be weighted to the effect of 10 points in the nomogram model score on the risk of TB mortality. Therefore, males have a higher risk of mortality, which may be due to their high labor intensity and social pressure, excessive smoking, alcohol consumption, irregular lifestyle, and poor resistance, while females have stronger health awareness. However, studies have also shown that there is no significant difference in the survival rates between genders [12]. In this study, the nomogram model shows that advanced age is a risk factor for the mortality of TB patients. With each year's increase in patients' age, the score of the nomogram model increased by 1 point, and the corresponding risk of mortality increased. Other research also shows that age is an important factor affecting the survival of TB patients; the reason may be that as age increases, the function of each viscera and the immunity of the organism will gradually decline, especially the elderly patients who have many complications, atypical symptoms, which may lead to delayed treatment or misdiagnosis [12, 13, 20, 21]. Furthermore, elderly patients may have to discontinue treatment for reasons such as adverse drug reactions, which further increase the risk of mortality.

In addition, the results of our study further suggest that tuberculosis relapse and first sputum bacteria positivity are independent risk factors of mortality in TB patients. Tuberculosis relapse will increase the weight of the effect of 3.9 points in the nomogram model scoring on the mortality risk, and first sputum bacteria positivity will increase the weight of the effect of 8 points in the nomogram model scoring on the mortality risk. Relapse cases are mostly caused by unreasonable chemotherapy. Compared with new cases, relapse cases are more complicated. Its treatment is more difficult and its risk of mortality is higher.

Coinfection with TB and HIV is a group of concomitant diseases that promote the progression and deterioration of lesions and rapidly lead to death, and one-fourth of AIDS patients die of tuberculosis. Several studies have shown that patients with HIV/TB coinfection have a higher risk of mortality than those with TB only [12, 22]. The result of our study shows that HIV positivity would increase the weight of the effect of 22.8 points in the nomograms model scoring on the risk of mortality, indicating that HIV/TB coinfection would accelerate the reduction of immune cell resistance. HIV would replicate and proliferate rapidly after entering the body, which increases the risk of TB morbidity and mortality. Studies have shown that early detection of HIV/TB dual infection patients and early administration of antituberculosis therapy can improve the efficacy of antiviral therapy, thereby reducing the fatality rate [23].

Our study also shows that patient delay is a risk factor for TB mortality. A delay of more than 14 days increases the weight of the effect by 3.8 points in the nomogram model scoring on the risk of mortality. Patient delay is caused by patients' insufficient attention to the suspected symptoms of TB, which directly results in the spread of lesions and exacerbation of the disease, which not only increases the burden of the disease but also affects the prognosis of patients. Delay in diagnosis and starting tuberculosis treatment increases the severity, risk of mortality, and transmission of the disease in the community [21]. Early detection and treatment of TB can improve the prognosis of patients and improve their quality of life.

In conclusion, our study establishes an individualized nomogram model to predict the risk of mortality in TB patients during DOTS based on the six risk factors of mortality, including male, advanced age, relapse cases, first sputum bacteria positivity, patient delay, and HIV positivity. The calibration curve shows that the mean absolute error of the predicted mortality risk by the model and the actual mortality risk is 0.003, and the ROC curve shows that the area under the curve of the predicted mortality risk by the model is 0.816, indicating that the prediction model has good differentiation and accuracy, and has high clinical application value, which is of guiding significance for the screening of high-risk groups of mortality and the formulation of intervention strategies.

There are also some limitations in our retrospective cohort study. The model in the present study lacks an effective external validation by using data from other settings, which may restrict the application of the nomogram. Another limitation owes to a lack of information on several potential confounders, such as BMI, smoking, alcohol use, and socioeconomic status. A review investigation shows that low income, low education, and alcohol abuse were associated with therapy failure. Similarly, low income and alcohol abuse were associated with MDR-TB [24]. Due to the economic limitations of the detection method, nutritional status of patients before antituberculous treatment, economic level, social support, liver, and kidney function levels, T-SPOT.TB, gene experts, and other factors are not included in the observation indicators. The outcome variable observed is all-cause mortality, and no distinction is made between deaths from TB and coincidental deaths from other causes. Since our study is a retrospective cohort study, no data on the extent of lung involvement, the severity of the disease at the beginning of the treatment, and the time of conversion of the sputum during the follow-up were collected. In the future, multicenter prospective studies with a larger sample size shall be carried out, and a more stable prediction model established with full consideration of relevant influencing factors.

## 5. Conclusion

The nomogram model based on independent risk factors of mortality in TB patients shows good discrimination and accuracy, with potentially high clinical value in screening patients with a high risk of death, which could be useful for setting the interventional strategies in patients with tuberculosis who had successfully initiated DOTS.

## Figures and Tables

**Table 1:** Patient clinical characteristics and statistical analysis of the death status.

Variable	Total cases (*N* = 11207)	Alive (*N* = 10697)	Died (*N* = 510)	*χ* ^2^ or *t*	*P* value
Age (years)	49.61 ± 19.99	48.66 ± 19.72	69.44 ± 14.58	30.851	<0.001^*∗∗*^
Sex					
Female	3421	3327 (31.10)	94 (18.43)	36.852	<0.001^*∗∗*^
Male	7786	7370 (68.90)	416 (81.57)		
Ethnicity					
Predominant group (Han)	11034	10533 (98.47)	501 (98.24)	0.172	0.697
Ethnic groups	173	164 (1.53)	9 (1.76)		
Case classification					
New	9107	8739 (81.70)	368 (72.16)	29.089	<0.001^*∗∗*^
Relapse	2100	1958 (18.30)	142 (27.84)		
First sputum bacteria results					
Negative/not tested	4794	4660 (43.56)	134 (26.27)	59.444	<0.001^*∗∗*^
Positive	6413	6037 (56.44)	376 (73.73)		
Pulmonary cavity					
No	8583	8209 (76.74)	374 (73.33)	3.153	0.076
Yes	2624	2488 (23.26)	136 (26.67)		
HIV status					
Negative/not tested	11187	10680 (99.84)	507 (99.41)	5.036	0.025^*∗*^
Positive	20	17 (0.16)	3 (0.59)		
Drug resistance results					
Nondrug resistance	9738	9285 (86.80)	453 (88.82)	1.750	0.186
Drug resistance	1469	1412 (13.20)	57 (11.18)		
Patient delay (d)					
No	7993	7650 (71.52)	343 (67.25)	4.320	0.038^*∗*^
Yes	3214	3047 (28.48)	167 (32.75)		
Diagnosis delay (d)					
No	8792	8387 (78.41)	405 (79.41)	0.292	0.589
Yes	2415	2310 (21.59)	105 (20.59)		

HIV, human immunodeficiency virus; ^*∗*^*P* < 0.05; ^*∗∗*^*P* < 0.01.

**Table 2:** Variable assignment table of logistic stepwise regression analysis.

Variables	Code	Variable assignment
Dependent variable		
Death	*Y*	0 = no
		1 = yes

Independent variable		
Sex	*X*_1_	1 = female
		2 = male
Age	*X*_2_	—
Ethnicity	*X*_3_	1 = predominant group (Han)
		2 = ethnic groups
Case classification	*X*_4_	1 = new
		2 = relapse
First sputum bacteria results	*X*_5_	0 = negative/not tested
		1 = positive
Patient delay	*X*_6_	0 = no
		1 = yes
Diagnosis delay	*X*_7_	0 = no
		1 = yes
Pulmonary cavity	*X*_8_	0 = no
		1 = yes
HIV status	*X*_9_	0 = negative/not tested
		1 = positive
Drug resistance results	*X*_10_	0 = nondrug resistance
		1 = drug resistance

**Table 3:** The multivariable unconditional logistic regression analysis of risk factors of mortality among pulmonary tuberculosis patients under the DOTS strategy.

Variables	*β*	SE	Wald *χ*^2^	*P*	OR	OR 95% CI
Sex	0.650	0.121	29.060	0.001	1.915	1.512∼2.425
Age	0.066	0.003	398.654	0.001	1.069	1.062∼1.076
Case classification	0.260	0.107	5.929	0.015	1.297	1.052∼1.599
First sputum bacteria results	0.537	0.107	25.305	0.001	1.711	1.388∼2.110
Patient delay	0.252	0.102	6.091	0.014	1.286	1.053∼1.571
HIV	1.504	0.659	5.202	0.023	4.497	1.235∼16.372
Constant term	−8.958	0.359	624.223	0.001	0.000	—

OR, odds ratio; CI, confidence interval; HIV, human immunodeficiency virus; SE, standard error.

**Table 4:** Nomogram scores of the risk of mortality among pulmonary tuberculosis patients under the DOTS strategy.

Variables	Nomogram scores
Age	1/year
Sex	10/male
HIV	22.8/positive
First sputum bacteria results	8/positive
Case classification	3.9/relapse
Patient delay	3.8/yes

## Data Availability

The datasets generated during and/or analyzed during the current study are not publicly available but are available from the corresponding author upon reasonable request.
